# Physical Literacy as A Framework of Assessment and Intervention for Children and Youth with Developmental Coordination Disorder: A Narrative Critical Review of Conventional Practice and Proposal for Future Directions

**DOI:** 10.3390/ijerph17124313

**Published:** 2020-06-17

**Authors:** Motohide Miyahara

**Affiliations:** Department of Clinical Psychological Science, School of Medicine, Hirosaki University, Aomori 036-8564, Japan; miyahara@hirosaki-u.ac.jp or motohide.miyahara@gmail.com

**Keywords:** physical literacy, developmental coordination disorder, norm-referenced test, criterion-referenced test

## Abstract

A framework of literacy may have roles to play in the assessment and treatment of children and youth with developmental disorders. This review aims to evaluate the conventional practice of assessment and treatment for children and youth with a developmental disorder in the physical domain, called developmental coordination disorder (DCD), and explore how the framework of physical literacy could contribute to the advancement of the current practice. This study adopts a method of narrative critical review based on a non-systematic search for its broad coverage to provide insights into the trend and future alternative directions. Over recent decades, children and youth with DCD have been typically assessed with standardized norm-referenced tests, before and after task-oriented intervention, for aiding diagnosis and evaluating the treatment effect. However, a recent high-quality systematic review showed limited evidence for the treatment effect assessed by the tests. Here, a framework of physical literacy is proposed to be used as an alternative to the conventional practice by recalibrating treatment goals and modifying the assessment and intervention approaches; criterion-referenced real-life authentic assessment and activities are encouraged with an emphasis on the enjoyment of movement and value of physical activity towards the attainment of physically active and healthy lifestyle goals from a lifespan perspective. The application of the physical literacy framework to the assessment and treatment of DCD needs to be further examined conceptually and empirically, while exploring a potential contribution of the literacy framework to transform the conventional assessment and treatment of children and youth with other developmental disorders.

## 1. Introduction

Neurodevelopmental disorders have a childhood onset and often persist into adulthood, requiring lifelong monitoring and support. To alter the developmental trajectories, early identification and intervention have been emphasized for individuals with neurodevelopmental disorders. For these individuals, the acquisition of basic skills or “literacy” is the primary focus; the development of literacy in the affected domains of reading, social communication, self-regulation, and physical activity have been considered appropriate as treatment goals of dyslexia [1], autism spectrum disorder [2], attention deficit hyperactivity disorder [3], and developmental coordination disorder (DCD) [4], respectively. The present review focuses on DCD, which affects 5–6% of school-aged children and features a significant delay in motor development with an early onset that interferes with activities of daily living in the absence of underlying neuromuscular or intellectual disability [5]. To enable effective identification and management of children with DCD, the appropriate assessment and intervention should be undertaken. Given the contemporary emphasis on evidence-based assessment and intervention for DCD [6], a critical review of assessment and intervention practices is timely and warranted; we need to identify and fill the gaps in implementing evidence into practice for individuals with DCD. The purposes of this review are to describe and critique in a constructive way the conventional practice of assessment and intervention for children and youth with DCD, and explore ways to narrow the gap between evidence and practice for both assessment and intervention. To align evidence and practice, the present investigation explores the utility of a framework of physical literacy to guide the assessment and intervention for individuals with DCD. 

Over the past two decades, children and youth with DCD have typically been assessed with standardized norm-referenced tests (elaborated later in Section 3.3.) and questionnaires, such as the Developmental Coordination Disorder Questionnaire [7], before and after intervention, for aiding diagnosis and estimating the treatment effect. However, a recent systematic review [8], the only review rated as high in quality to date [9], showed limited evidence for the treatment effect assessed by a norm-referenced test. The questionnaires were not included in the systematic review as outcome measures because they had not been used as frequently as the tests for evaluating the intervention effects [8], and therefore they are not discussed in this review. Since the assessment of intervention efficacy with norm-referenced motor tests has demonstrated to be unfruitful, it is important to consider an alternative framework, such as physical literacy, to guide assessment and intervention practice for children with DCD.

Physical literacy is a metaphorical use of the term literacy—the ability to read and write in the language domain—in the context of the physical domain. In parallel with the learning process of reading and writing, the process of acquiring physical literacy harnesses children’s potential not only in developing knowledge and skill that are required to function in their cultures and enable them to participate in their societies; development of physical literacy is also concerned with quality of life which encompasses health-related values and personal responsibilities [10]. These aspirations are achieved through intrinsic motivation elicited by exposing children to a wide variety of physical activities in different environments, incorporating their development in the cognitive, psychosocial [11], and spiritual [12] domains from a lifespan perspective [13]. A physical literacy assessment and curriculum currently exist for a general population, such as the Canadian Assessment of Physical Literacy [14,15]. However, to the author’s knowledge, a framework of physical literacy has not been applied to the assessment and intervention of DCD.

## 2. Materials and Methods

Materials used in this review are published studies identified by the author, including journal articles, book chapters, and test manuals. As a method of search strategy, the author has opted out of a systematic search, in favor of a non-systematic search, to cover a broad range of sources [16,17,18] which suitably enables the author to identify the trend [19], gain in-depth insight [18,19,20], engage in critical reflection [18], and offer a new model application [18] of physical literacy for a future solution in the uncharted territory [21]. Adopting a method of expert-led narrative critical review for the analysis, evaluation, and interpretation of the literature [18,20], this review study first describes and evaluates the prevailing assessment and intervention practices for children and youth with DCD from the current standard of evidence-based assessment and intervention. Secondly, the framework of physical literacy is presented, highlighting the concepts of minimum proficiency, criterion-referenced, and authentic assessment which have been overlooked or unexplored. Finally, implications for future assessment and intervention practices, using the framework of physical literacy, are discussed.

## 3. Literature Review Sections

### 3.1. A Typical Set of Assessment and Intervention Procedures: The Conventional Model

Among researchers, as well as practitioners, it has been a common belief that task-oriented intervention is effective for children and youth with DCD and that the intervention effect can be inferred by observing differences between pre- and post-test results of a norm-referenced assessment. Task-oriented intervention carefully aligns specific movement tasks for practice and mastery. For example, if you want a child with DCD to learn how to tie shoelaces, the task of shoelace tying is practiced with verbal guidance until the skill is mastered. When did the task-oriented intervention appear in the research literature as a treatment for individuals with DCD? How was the belief in the significant effects of task-oriented intervention on norm-referenced tests formed? What sort of evidence exists to support the claim? 

A meta review or umbrella review (i.e., a review of systematic reviews) conducted in 2017 [22] historically traced back the primary intervention studies from 1971 till 2011 that had been cited by four systematic reviews published between 1996 and 2013. Among the primary intervention studies, Revie and Larkin’s (1993) task-specific intervention study [23] is the first published research report comparing intervention efficacy against a matched active control group of children who were perceived as poorly coordinated by their teachers. Note that the matched control group was not randomly generated and assigned. In this study, the effects of specific task-teaching for the skills of distance throw, distance hop, target kicking, and volleyball bounce and catch were measured correspondingly by distance, accuracy score, or number of successful trials. It is also worth drawing attention to the fact that this pioneering study’s use of a norm-referenced test was limited to the confirmation of incoordination in the participants, and not for evaluating the intervention effects. Such an assessment practice is consistent with the authors’ theoretical position that undermines skill transfer from the practiced tasks into the test tasks [24].

Of the early intervention studies narratively reviewed by Polatajko and Cantin in 2005 [25], the task-oriented intervention study conducted by Wright and Sugden in 1998 [26] is the first published trial that employed a norm-referenced test and questionnaire to examine the intervention effects. Using both assessment tools, school teachers conducted task-oriented intervention to children with DCD and significantly improved the scores of both types of assessment after the intervention. Ever since, a series of task-oriented intervention studies (e.g., [27,28,29,30,31]) has routinely employed norm-referenced assessment tools to evaluate and report significant effects of task-oriented intervention. 

### 3.2. Limited Evidence for the Prevailing and Recommended Practice for Assessment and Intervention

A great majority of the studies that investigated the efficacy of task-oriented intervention did not use a randomized control trial (RCT) design, but relied on non-RCT designs [8]. When comparing pre- and post-intervention assessment results, non-RCT designs are considered to control less confounding and increase the possibility of alternative explanations for the intervention effect than RCT designs [32]. Due to the extraneous factors blurring the true intervention effect in non-RCT studies [33], the estimates of intervention effects from the non-RCT studies have not been regarded as credible as those from RCT studies [34]. 

What are the estimates of task-oriented intervention effects by RCT studies, and how are they different from the estimates made by non-RCT studies? To date, there is only one systematic review [8] that exclusively covered RCTs and quasi RCTs, conducted meta analyses, and computed estimates of task-oriented intervention effects on a common norm-referenced test. The review found that some trials had produced estimates of significant intervention effects, but that the effects could not be ascertained until higher quality, or more carefully designed and conducted RCTs become available. In short, contrary to the prevailing view of significantly positive intervention efficacy, which may or may not be replicable in further RCTs, the currently available high-quality evidence is still too limited in quantity and quality to conclude any definite effect of task-oriented intervention [9]. Nonetheless, the most recent international clinical recommendations for DCD [6] suggest that a norm-referenced assessment should be administered to monitor progress at least every three months to evaluate the recommended interventions which should be task/activity-oriented and participation-oriented. 

### 3.3. Norm-Referenced Test, Its Tenets and Malleability

#### 3.3.1. What Norm-Referenced Tests Are Suitable and Unsuitable for

A norm-referenced motor assessment provides information regarding motor function of an individual in a normative sample [35]. Some of the popular assessment tests for children and youth with DCD include the Movement Assessment Battery for Children (MABC) and its second edition, MABC-2 [36,37], and the Bruininks–Oseretsky Test of Motor Proficiency (BOT) and its second edition, BOT-2 [38,39]. These performance outcome tests are regarded as motor ability tests [24,40]. The ability tests purposefully employ decontextualized, neutral movement tasks for test items, so that all test takers should perform each test item with an equal familiarity with no advantage to anyone [41]. Due to this nature of ability tests, a norm-referenced assessment is suitable to identify where an individual is placed in the population standard. In the case of DCD, the current norm-referenced tests [37,39] yield percentile values which are interpreted to determine whether or not a test taker meets the diagnostic cutoff. Usually, scores ranging from the 1st to 15th percentile indicate DCD. Thus, norm-reference tests may be useful for identifying and diagnosing a child with DCD.

Is it appropriate to use norm-referenced tests for monitoring and evaluating intervention outcome? To answer this question, it is worth considering the implications for the intervention outcome linked with the norm-referenced tests. Recall that the norm-referenced tests are construed as ability tests, and note the definition of ability, that is, “a fundamental characteristic of different individuals that tends to underlie particular skills; ability is largely inherited genetically and is not modifiable by practice” [42] (Schmidt and Lee, 2013, p. 151). In the definition of DCD characterized by a significant delay in motor development which interferes with activities of daily living, the significant delay implies a shortcoming of the ability to execute skills that are required to perform specific motor tasks. Provided that children with DCD perform norm-referenced tests at the lower end of the continuum, the test tasks are obviously too difficult or even inappropriate for the children’s ability levels to work on improvement [43]. In addition, abilities are relatively stable and enduring traits by definition; the motor ability test scores should not be malleable in a short time. Drawing on a study that successfully demonstrated a significant effect of early intervention on IQ from early childhood into young adulthood (i.e., 3, 4, 5, 6.5, 8, 12, 15, 21 years) [44], the author proposed to leave a minimum gap of one year between consecutive motor ability testing time points. With more than a year of gap, the test taker is compared to an older age norm every time, possibly by being tested with more advanced different test items, making it difficult to “practice for the test”. 

If the clinical significance of the intervention outcome relies on improvement in motor ability test scores beyond the diagnostic cutoff, there may be a problem with either (1) the stability of the motor ability tests; (2) the intervention goal; or (3) the choice of the tests for assessing intervention outcome. 

#### 3.3.2. Stability and Responsiveness of the Motor Ability Tests Scores

The stability of tests can be operationally defined in terms of *test–retest reliability,* as opposed to malleability or *responsiveness to change* as a result of intervention. Standardized developmental motor ability tests, such as the MABCs and BOT-2, demonstrate good test–retest reliability (0.73–0.84; 0.52–0.95) [36,45,46]. This means that the test scores are temporally stable over short periods of time without any intervention. If the tests are used for monitoring and evaluating the efficacy of intervention, the test scores need to be responsive enough to intervention effects. To reflect the responsiveness, a *minimal detectable change* (MDC) is computed by considering the variability (instability) of the test scores without any intervention, with the computed MDC value indicating a required magnitude to determine the intervention effect [47]. To date, MDC has been examined only with the MABC and MABC-2 [48,49].

In the case of the first edition of the MABC, the MDC for the total MABC score is 8.68 at the 95% confidence interval [48]. This means that a change of equal to or greater than 8.68 would be required to consider that a change between the before and after intervention derived from the intervention, not from the instability of the test. Among the RCTs and quasi RCTs systematically reviewed and meta-analyzed [8], Tsai et al.’s (2012) trial [50] demonstrated the largest effect size, yet the difference between the before and after intervention was smaller than 8.68; the magnitude of the change in the MABC scores over the intervention period is better explained by chance than as a result of the intervention. With the MDC considered, the responsiveness of the MABC falls short of detecting even the largest intervention effect documented in the literature.

In its second edition, the MABC-2, the scoring system has changed from the MABC test score, that was associated with greater motor impairment, to the MABC-2 standard score with the mean of 10 and SD of 3. So far, there is only one study by Wuang et al. (2012) [49] that evaluated the responsiveness to change for a DCD sample on the MABC-2. This study first computed the variance of 139 children whose mean MABC-2 total standard score was 8.0 (25th percentile) at the initial visit. Twenty days later, the children were re-tested, and based on the test–retest variance, an MDC was computed after rehabilitation programs over six months. While the mean change after the intervention was 1.30 (SD = 2.10, Effect Size (ES) = 0.42), increasing the mean total standard score to 9.3 (41st percentile), the MDC was 1.21 at the 90% confidence interval. Thus, the change after the intervention was larger than the MDC, demonstrating a valid change. Although this pioneering study on responsiveness appears to succeed in the intervention with a small-medium ES and in demonstrating the responsiveness of the MABC-2, there are two major limitations in this study. First, the sample’s MABC-2 mean total standard score of 8.0 (SD = 3), or on the 25th percentile, is relatively high compared with the common upper limit of the diagnostic cutoff point, the 16th percentile. Therefore, findings from this study may not be applicable to the DCD population in general. Second, the non-intervention period between test and retest was 20 days, while the intervention period was six months. The MDC based on the variance over the 20 days might underestimate a potential variance over six months, allowing the detection of intervention change more easily. These limitations make the interpretation of the MABC-2’s responsiveness to change questionable. Taken together, the two common motor ability tests have demonstrated excellent stability but not responsiveness, which match the definition and mission of motor ability tests to place an individual in an age-norm standard.

#### 3.3.3. Setting an Intervention Goal to Improve Beyond the Cutoff Point of Norm-Referenced Tests

The Jacobson–Truax method for assessing clinically meaningful change [51] sets a cutoff point as a threshold to overcome; thereafter, a person who was first placed in a clinical group transits to outside the range of the clinical group after intervention. Clinical significance is mentioned when an intervention results in the removal of the disorder or diagnosis [52]. If this method is applied to the intervention goal of children with DCD, they may attempt to improve a norm-referenced test score beyond the cutoff. If successful, they are no longer classified as having DCD. There are two problems with applying this method to DCD intervention. One is that clinically meaningful change has not been established or even discussed for children with DCD. The other problem is concerned with goal-setting. 

To the author’s knowledge, there are only a few studies that have dealt with clinically meaningful change in norm-referenced test scores for children with DCD. First, the simplest way to delineate clinically meaningful change is to take a diagnostic cutoff point (e.g., 15th percentile) for granted, and accept any changes across this cutoff from below to above as clinically meaningful changes [8,31]. However, this approach is open to question as to the validity of the cutoff point that reflects clinical significance [53]. Second, while subjectivity inescapably comes into play in clinical significance due to variation in clinical goals and interventionists’ experiences, the effect size is considered as the most important indicator of clinical significance [54]. In line with this assertion, a systematic review conducted by Preston et al. [55] pointed out that a ten-week table tennis training by Tsai (2009) [56] is statistically significant, but “the effect is negligible in terms of its clinical significance” (p. 864) without defining clinical significance. For example, a child with DCD, who improved with a small effect size on the norm-referenced test, might acquire not only table tennis skills, but also a leisure skill to enjoy table tennis for the rest of life. As a result, the small effect size can be perceived as beneficial and clinically significant. This is an example of external validation of norm-referenced test score changes. 

Such validation of responsiveness can be computed and named a *minimal clinically important difference* (MCID) [54] which is defined as the smallest difference in scores which the test taker perceives as beneficial [57]. In Wuang et al.’s (2012) study introduced earlier, the changes in the MABC-2 score were externally validated with therapists’ ratings on the children’s performance of school-related physical tasks as to whether they improved or not after the intervention programs [49]. The MCID of the MABC-2 score was 2.36, larger than mean change of 1.3 (SD = 2.1) after the intervention, indicating that the intervention was unable to achieve an MCID for a majority of the children with DCD. If the numerical goals need to be verified by subjective ratings of improvement, why not have the child with DCD or the therapist set the goal to start with? Is the dichotomous rating of “improved” or “no change” sufficient for monitoring and evaluating intervention?

In addition to the problem of associating the cutoff point with clinical significance, setting an intervention goal based on a norm-referenced test score is also problematic from a pedagogic point of view. Such an intervention practice has been construed as “teaching to the test”. It is considered pedagogically unsound because the practice can narrow curriculum [43] and limit the applicability of learning to a real-life functional context [58] due to the nature of neutral, decontextualized test items. The problem lies in the disconnect between the neutral and decontextualized test items and specific and contextualized teaching objectives. Instead of focusing on raising test scores, a task-oriented intervention called an *ecological intervention program* views participation as a starting point and emphasizes setting intervention objectives, focused on functional tasks that are enjoyable, meaningful, and relevant to the child and significant others [59]. This principle is consistent with the tenets of physical literacy which emphasize positive movement experiences to foster life-long participation in health-related physical activity by adapting environment, equipment, rules, and assistant services [60,61]. However, the attainment of such behavioral objectives may not always reflect changes in norm-referenced test scores [41]. How would it be possible to monitor and evaluate the progress and effect of intervention without using norm-referenced tests?

### 3.4. Alternative Assessment and Intervention Strategies in the Framework of Physical Literacy: The Proposed Model of ‘Assessing with and Teaching to Contextualized Criterion-Referenced Tests’ to Meet the Minimal Proficiency

In discussing physical literacy and individuals with a disability, Vickerman and DePauw (2010) maintain that “alternative methods of charting progress which maximize opportunities for all to demonstrate growing physical literacy should be established” (p. 135) [62]. As alternatives to using norm-referenced assessment for monitoring and evaluation, the author proposes a criterion-referenced assessment of task-oriented intervention. This would link assessment with intervention by setting step-by-step observable behavioral objectives from the presently attainable task to the mastery of an ultimate goal task (see Table 1 for an example). In a long-standing textbook of developmental physical education, Donnelly, Mueller, and Gallague (2017) state: “Teachers can enhance children’s motivation by encouraging them to set criterion-referenced, task-oriented goals that focus on personal improvement and mastery of skill.” (p. 25) [63]. The textbook authors also point out that learners’ focus on realistic and personal criterion-referenced objectives could discourage upward social comparisons and the resultant sense of inferiority. The provision of such an individualized set of objectives can form a part of Vickerman and DePauw’s (2010) “strategies to support physical literacy [for children with disabilities that] should be carefully planned, focused, and have a clear purpose of offering opportunities to experience success and satisfaction” (pp. 134–135) [62]. Personal success and fulfilment in turn offer children with opportunities to appreciate being of value and responsible [10]. When a child with DCD attempts an item in a standardized norm-referenced test, such as a task of catching a tennis ball from a distance, the child may not be able to succeed in any trials of the task at all. Although such a test item may entertain children in the middle range and potentially bore the upper end of the norm, the item may be inappropriate for the child with DCD. This poses a risk of demotivating the child to participate in intervention following the assessment. By contrast, a criterion-referenced assessment allows the child and the interventionist to set realistically achievable minimum proficiency goals.

The notion of literacy implies that *minimum proficiency* would be sufficient for taking advantage of learned skills—whether reading, writing, math [65], or physical skills [66]—for enriching the entire life. In addition to skill learning, the development of literacy involves exposure to and participation in a wide range of materials, contexts, and environments, mobilizing cognitive, emotional, social, and spiritual resources from a lifespan perspective. For example, the Assessment of Physical Literacy in Physical Education: Passport for Life in Canada uses a scale of “emerging”, “developing”, “acquired”, and “accomplished”, and aims for the general population to attain the “acquired level” [15]. However, the acquired level for typically developing children may not be a suitable goal for children with DCD. The following sections first explain the criterion-referenced assessment, then provide a few examples of criterion-referenced tests relevant to children with DCD, while delineating its limitations. Finally, outstanding challenges for the use of a criterion-referenced assessment, and areas for future research and development will be discussed.

#### 3.4.1. What Criterion-Referenced Assessment Is Suitable and Unsuitable for 

Unlike a norm-referenced assessment, a criterion-referenced assessment, by itself, has no age norm and is not designed to place an individual in a normative sample. Instead, the assessment consists of criteria of task performance which are derived from a task analysis [35]. As obvious from the repetitive use of the word task in its definition, a criterion-referenced assessment is task-oriented and suitable for planning, monitoring, and evaluating task-oriented interventions. The connection between the assessment and intervention tasks in the real-life, contextualized, and meaningful environment enables authentic learning [67]. Individual performance is compared to an achievement continuum of developmental criteria within a set period of time [68]. The achievement continuum may be either consistent with objectives of intervention sessions tailored to an individual child (e.g., Table 1), or comprised of a Likert scale rating how well each criterion is performed, for example, “poor”, “satisfactory”, or “good”. In either case, a criterion-referenced assessment encourages reflection of the present performance and individual progress without comparing the individual performance against a cohort [69]. 

Considering the categories of quantitative decision-support evidence and qualitative knowledge-support evidence developed by Pope, Mays, and Popay (2006) [70], a criterion-referenced assessment is more suitable to provide evidence for knowledge support (i.e., how to support a child with DCD to perform a particular task under a specific condition) than evidence for decision support. (i.e., how effective task-oriented intervention is). Although it is not the inherent nature of the criterion-referenced assessment to provide quantitative evidence for group data, there have been a few attempts to do so. The few exemplar tests seem to contain seeds for future development of a unified quantitative index derived from the criterion-referenced assessment. Brief descriptions of and implications from the tests for the solution will be laid down in the following sections.

#### 3.4.2. Criterion-Referenced Standardized Tests

In the field of education, tests can be categorized into high-stakes vs. low-stakes tests. A high-stakes test has personal consequences for test takers [71] and is used to make crucial decisions, such as school entry and graduation; fairness to all test takers is essential for high-stakes tests. A low-stakes test measures academic achievement, identifies learning problems, or informs instructional adjustments [72]. In the context of clinical management of DCD, diagnostic tests can be considered high-stakes tests, and daily clinical and teaching assessment to monitor and evaluate intervention outcome may be regarded as low-stakes tests. To meet the fairness obligations of high-stakes tests, most diagnostic tests use standardized procedures and fixed test items, which is incongruent with the nature of a tailor-made criterion-referenced assessment for specific tasks. Nonetheless, three standardized tests, namely the Test of Gross Motor Development (TGMD), the Brockport Physical Fitness Test (BPFT), and the Performance Quality Rating Scale (PQRS) embody the structure of a criterion-referenced assessment.

##### TGMDs

The Test of Gross Motor Development (TGMD) and its second and third editions, TGMD-2 and TGMD-3 [73,74,75], use a form of criterion-referenced approach to assess locomotor and object control skills. Each test item consists of a set of 3–5 criteria for movement patterns associated with skill proficiency. Test takers’ performance of each skill is analyzed by the composite approach of developmental biomechanics, in that movement patterns of primary body parts are qualitatively analyzed and categorized [76]. The environment, equipment, and instructions for the test administration are specifically prescribed. Each of the criteria is rated by the tester on the scale of 1 (criterion fulfilled) or 0 (criterion unfulfilled). In addition to the foundation of the criterion-referenced assessment of movement patterns, the TGMDs yield a motor quotient (MQ). Like IQ, MQ is based on the total scores of all subtests in the age-norm standards. This aspect is clearly not criterion-referenced, but norm-referenced by placing a test taker in the normative standard. Thus, the TGMDs incorporate both criterion-referenced and norm-referenced procedures [40]. 

Note that the criterion-referenced aspect of the TGMDs is not based on a linear continuum of developmental progression as in the case of a conventional tailor-made criterion-referenced assessment. Probably for this reason, the test items of the TGMDs have been studied as task goals only once [77], which happened to be in the context of DCD intervention. Although the TGMDs have been used for other DCD intervention studies (e.g., [78]), there has been no research examining the minimal detectable change (MDC) and minimal clinically important difference (MCID) of the TGMDs for the DCD population to date [46]. The most important implication of the TGMDs lies in the invention of MQ that converts criterion-referenced ratings to quantitative norms.

##### The Brockport Physical Fitness Test (BPFT)

The BPFT [79] assesses health-related criterion-referenced aerobic functioning, body composition, and musculoskeletal functioning of individuals with a specific disability (i.e., blindness, intellectual disability, cerebral palsy, spinal cord injury, or congenital anomaly/amputation). It evaluates whether the test taker meets either the “minimal standard” or “preferred standard” for each test item. There is no disability category for DCD, therefore the BPFT has never been applied to the DCD population. However, some of the BPFT’s principles would be useful for the future development of DCD assessment and intervention. Firstly, the BPFT distinguishes skill fitness and physical fitness, and focuses not on skill fitness, but on physical fitness. This is probably another reason why the BPFT has never been applied to the DCD population. Second, the BPFT strives for health related fitness, not elite performance, with the goal of attaining the minimal standard for health and potentially further into the preferred standard. The health-related goal, comparison within the same disability group, and the realistic goal achievement of the minimal criterion-referenced standards are consistent with the tenets of physical literacy, with a potential application to DCD assessment and intervention.

##### The Performance Quality Rating Scale (PQRS)

The Performance Quality Rating Scale (PQRS) [80] is administered by occupational therapists who observe actual or video-recorded performance and rate on observable and operational criteria for specific activities (i.e., tasks) on a scale of 6 (0: not attaining activity criteria to 5: meeting all activity criteria; performance or product were of good quality) [80] or 10 (1: cannot perform the task at all to 10: can perform the task very well) [28] points. Originally developed for children with DCD, the PQRS has been used to monitor the steps (i.e., progress) and quality of performance towards goals selected by children with DCD. To detect a meaningful change, a score change of 3 or greater is deemed necessary, given the scale’s inherent variability [81]. The strength of PQRS in directly assessing specific tasks that children with DCD practice on is also its weakness in its limitation to synthesize intervention effects across individuals on different activities and tasks.

## 4. Conclusions

The present review described and critiqued the conventional model of monitoring and evaluating task-oriented intervention by norm-referenced tests, and proposed the development and use of individualized criterion-referenced tests. Given the limited evidence for the intervention efficacy evaluated with norm-referenced motor tests, the author applies the tenets of physical literacy, and proposes the following recommendations for the assessment and intervention practice for children with DCD:

1. Administer norm-referenced tests for diagnosis, not for monitoring and evaluating intervention in the short-term;
2. If a child with DCD is to be retested with a norm-referenced test, the time duration between two tests should be at least one year;
3. Monitor progress and evaluate the effect of an intervention program with a clinician- or teacher-generated criterion-referenced test that is tailored to a particular child with DCD;
4. Shift the intervention focus from enhancing the motor ability test score to (1) acquisition of the minimum proficiency to attain a functional task; (2) positive physical and movement experiences that could underpin life-long enjoyment of a healthy active lifestyle; and (3) participation in family, school, and community activities by adapting rules and structures of activities, employing compensatory equipment and assistant services.

Outstanding research challenges include the production of high-quality evidence that qualitatively and quantitatively demonstrates the progress and effect of intervention with criterion-referenced tests. Future qualitative research should describe the process and outcome of individualized progressive intervention, and provide insights into how and why objectives and associated criteria for success are developed, modified, and attained. An innovative development is necessary to transform the criterion-referenced test results across different settings into a unified quantitative index. Finally, more critical narrative reviews are needed to play complementary roles for systematic reviews and apply a new model, such as the literacy framework, to DCD and other developmental disorders.

## Figures and Tables

**Table 1:** An example of step-by-step observable behavioral objectives with exemplary criteria of success (Step 1–3) entitled, “You can bike!” from the Family Focused Tele-Intervention for Children with Developmental Coordination Disorder [64].

**Step 1:** Stand over the bike ^1^	**Step 2**: Sit on the bike ^2^	**Step 3**: Put weight on the seat ^3^	**Step 4**: Shift weight from side to side while standing	**Step 5**: Shift weight side to side with feet on the ground	**Step 6**: Lean side to side, lifting the feet off the ground	**Step 7**: Glide on a flat surface
**Step 8:** Use the break to decelerate and stop	**Step 9:** Glide on a gentle slope	**Step 10:** Put the feet on the pedals	**Step 11:** Pedal the bike	**Step 12:** Put one foot on the pedal on a slope	**Step 13:** Steer the handle bar	**Step 14:** Put one foot on the pedal and push on a flat surface

^1^ Criteria of success for Task 1: Stand over the bike over the seat which is low enough to go between the legs of the child for 10 s; ^2^ criteria of success for Task 2: From the Task 1 position, sit on the saddle by bending the knees with both feet on the ground and the hands holding the handle bars. If the bottom touches the seat, that is considered as success; ^3^ criteria of success for Task 3: Start from the standing position over the bike, feet on the ground and hands holding the handle bars. Bend the knees and sit on the bike seat with the feet on the ground for 10 s.
